# Pla2g6 Deficiency Induces Neuronal Precursor Apoptosis During Neurodevelopment

**DOI:** 10.3390/ijms27104280

**Published:** 2026-05-11

**Authors:** Yang-Jin Shen, Han-Fang Liu, Ting-Chen Hsu, Yi-Chieh Chen, Yi-Chuan Cheng

**Affiliations:** 1 Graduate Institute of Biomedical Sciences, College of Medicine, Chang Gung University, Taoyuan 333323, Taiwan; 2 Neuroscience Research Center, Chang Gung Memorial Hospital, Linkou, Taoyuan 333423, Taiwan; 3 Department of Neurology, Chang Gung Memorial Hospital, Linkou, Taoyuan 333423, Taiwan

**Keywords:** Pla2g6, neurogenesis, neuronal precursors, apoptosis, oxidative stress, parkinsonism

## Abstract

Phospholipase A2 group VI (PLA2G6) regulates phospholipid remodeling and cellular homeostasis, and its mutations cause neurodegenerative disorders, including neurodegeneration with brain iron accumulation and PLA2G6-associated parkinsonism (PARK14). Although many cases present in adulthood, a substantial subset shows early onset, indicating that PLA2G6 dysfunction can affect neuronal systems during developmental stages. However, whether PLA2G6 directly regulates early neurogenesis remains undefined. Here, using zebrafish embryos, we investigated the role of Pla2g6 during neural development through loss- and gain-of-function approaches. *pla2g6* is dynamically expressed during embryogenesis, with enrichment in the developing central nervous system during neurogenesis. CRISPR/Cas9-mediated Pla2g6 deficiency did not alter neural progenitor formation but significantly reduced neuronal precursors. Expression of the disease-associated PLA2G6 D331Y variant phenocopied this effect, confirming that the observed phenotype results from loss of Pla2g6 function. The reduction in neuronal precursors occurred without changes in proliferation but was accompanied by a marked increase in apoptosis, identifying neuronal precursor cell death as the primary mechanism. Under oxidative stress conditions, Pla2g6 overexpression reduced neuronal apoptosis, whereas Pla2g6 deficiency markedly enhanced reactive oxygen species -induced apoptosis. These findings establish Pla2g6 as a regulator of oxidative stress-associated apoptotic signaling during neurogenesis. Together, these results define Pla2g6 as a stage-specific determinant of neuronal precursor survival, linking lipid homeostasis and oxidative stress control to early neural development. This study establishes a developmental framework for PLA2G6-associated disorders and positions impaired neuronal precursor survival as a contributing mechanism underlying disease onset.

## 1. Introduction

Phospholipase A2 group VI (PLA2G6), also known as calcium-independent phospholipase A2β (iPLA2β), catalyzes the hydrolysis of phospholipids to generate free fatty acids and lysophospholipids, thereby contributing to phospholipid turnover and lipid remodeling [1,2]. This enzymatic activity is Ca^2+^-independent and operates in both cytosolic and membrane-associated compartments, reflecting a role in dynamic regulation of cellular lipid composition [3,4]. In addition to its catalytic function, PLA2G6 contains ankyrin repeat domains and exists in multiple alternatively spliced isoforms that modulate its activity, indicating regulatory functions beyond phospholipid hydrolysis [3,5]. Through the generation of lipid-derived signaling molecules and regulation of phospholipid homeostasis, PLA2G6 participates in diverse cellular processes, including cell cycle progression, proliferation, and stress-associated signaling pathways [6,7]. These combined biochemical and regulatory properties position PLA2G6 as a mediator linking lipid metabolism to cellular homeostasis and viability.

Mutations in PLA2G6 are associated with a variety of neurodegenerative disorders. A major subset of these belongs to neurodegeneration with brain iron accumulation (NBIA), a group of disorders characterized by iron deposition in the basal ganglia, within which PLA2G6-related disease is classified as NBIA type 2, including infantile neuroaxonal dystrophy (INAD; NBIA2A) and atypical neuroaxonal dystrophy (NBIA2B) [8,9,10]. In addition to NBIA-associated phenotypes, PLA2G6 mutations also cause dystonia–parkinsonism (PARK14), which may occur with or without detectable brain iron accumulation [11,12]. Functional analyses indicate that PLA2G6 mutations associated with NBIAs markedly impair enzymatic activity, whereas PARK14-associated mutations do not uniformly disrupt catalytic function [13]. These observations distinguish the biochemical effects of mutations underlying different PLA2G6-associated phenotypes. In parallel with this molecular heterogeneity, PLA2G6-related disorders show a broad age range at onset rather than a strictly adult presentation. The occurrence of childhood-onset phenotypes across this disease group indicates that PLA2G6 dysfunction can affect neuronal systems during developmental stages.

The presence of childhood-onset phenotypes raises the question of how PLA2G6 regulates early neural development. Neural development requires tightly coordinated regulation of cellular differentiation, survival, and proliferation, processes that depend on precise control of intracellular signaling and cellular homeostasis. *PLA2G6* is expressed in neural progenitor regions of the developing brain, including ventricular zones associated with neurogenesis [14]. These observations place PLA2G6 within cellular contexts relevant to neurogenesis, yet its role in specific steps of neural lineage progression has not been defined. To address this question, the present study investigates the role of Pla2g6 in zebrafish embryonic neural development. Using loss-of-function and gain-of-function approaches, this study examines how Pla2g6 influences neuronal precursor formation and cell survival during early neurogenesis. These analyses define the developmental stage and cellular context in which Pla2g6 function is required and establish a framework linking PLA2G6-associated disease phenotypes to processes operating during neural development.

## 2. Results

### 2.1. Zebrafish pla2g6 Is Dynamically Expressed in the Developing Nervous System

**Figure 1:**
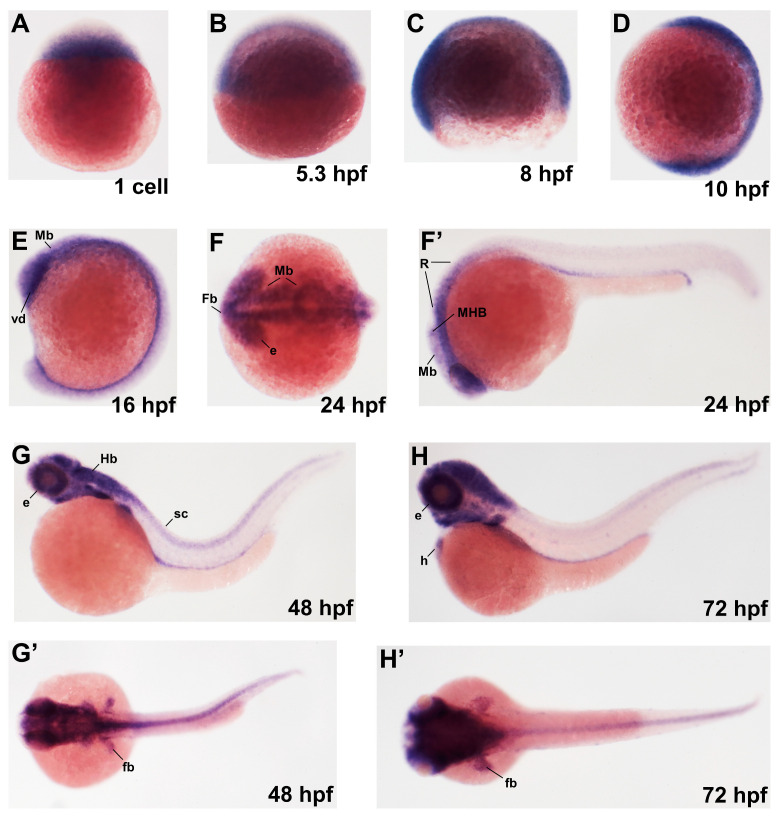
Dynamic expression of *pla2g6* during zebrafish embryonic development. Whole-mount in situ hybridization analysis showing the temporal and spatial expression pattern of *pla2g6* during zebrafish embryogenesis. Embryos are shown in lateral or dorsal views with anterior to the left and dorsal to the top, unless otherwise indicated. (**A**) Maternal *pla2g6* transcripts were detected at the one-cell stage. (**B**–**D**) During blastula and early gastrula stages (5.3–10 hpf), *pla2g6* expression was broadly distributed throughout the embryo. (**E**) At mid-segmentation stages (16 hpf), expression became enriched in the developing neural plate, including the presumptive brain and spinal cord. (**F**,**F’**) By 24 hpf, strong expression was observed in the developing brain and developing foregut and hindgut. (**G**,**G’**) At 48 hpf, *pla2g6* expression was predominantly localized to brain, with detectable expression along the spinal cord. (**H**,**H’**) At 72 hpf, expression remained enriched in the brain, with additional signals detected in the developing heart and fin buds. Abbreviations: Mb, midbrain; Fb, forebrain; Hb, hindbrain; MHB, midbrain–hindbrain boundary; sc, spinal cord; e, eye; h, heart; vd, ventral diencephalon; R, rhombomere.

Zebrafish offer several experimental advantages for studying neural development, including external fertilization, rapid embryogenesis, and optical accessibility of the developing nervous system. To characterize the temporal and spatial expression pattern of zebrafish *pla2g6* during embryogenesis, we isolated the zebrafish *pla2g6* and performed whole-mount in situ hybridization across key developmental stages. *pla2g6* mRNA were detected at the one-cell stage, indicating maternal contribution. During the cleavage stages [0.75–2.25 h post-fertilization (hpf)] and blastula period (2.25–5.25 hpf), *pla2g6* expression was broadly distributed throughout the embryo. Beginning at late blastula stages, expression became progressively enriched in neural tissues. By the bud stage (10 hpf), strong *pla2g6* signals were evident in the presumptive brain and spinal cord primordia (Figure 1). During mid-segmentation stages (16 hpf), *pla2g6* expression became restricted to the developing brain, with comparatively weaker signals detected in the spinal cord. At 24 hpf, *pla2g6* expression was observed throughout the brain, while spinal cord expression remained low. Additional expression was detected in the developing gut at this stage (Figure 1). Following the pharyngula period (48 hpf), *pla2g6* expression was predominantly localized to the central nervous system, with additional signals observed in the developing heart and fin buds (Figure 1). Together, these results demonstrate that zebrafish *pla2g6* is dynamically regulated during embryogenesis, with progressively enriched expression in the central nervous system. This expression pattern suggests a potential role for pla2g6 in neural development.

### 2.2. Loss of Pla2g6 Selectively Reduces Neuronal Precursors but Not Neural Progenitors

**Figure 2:**
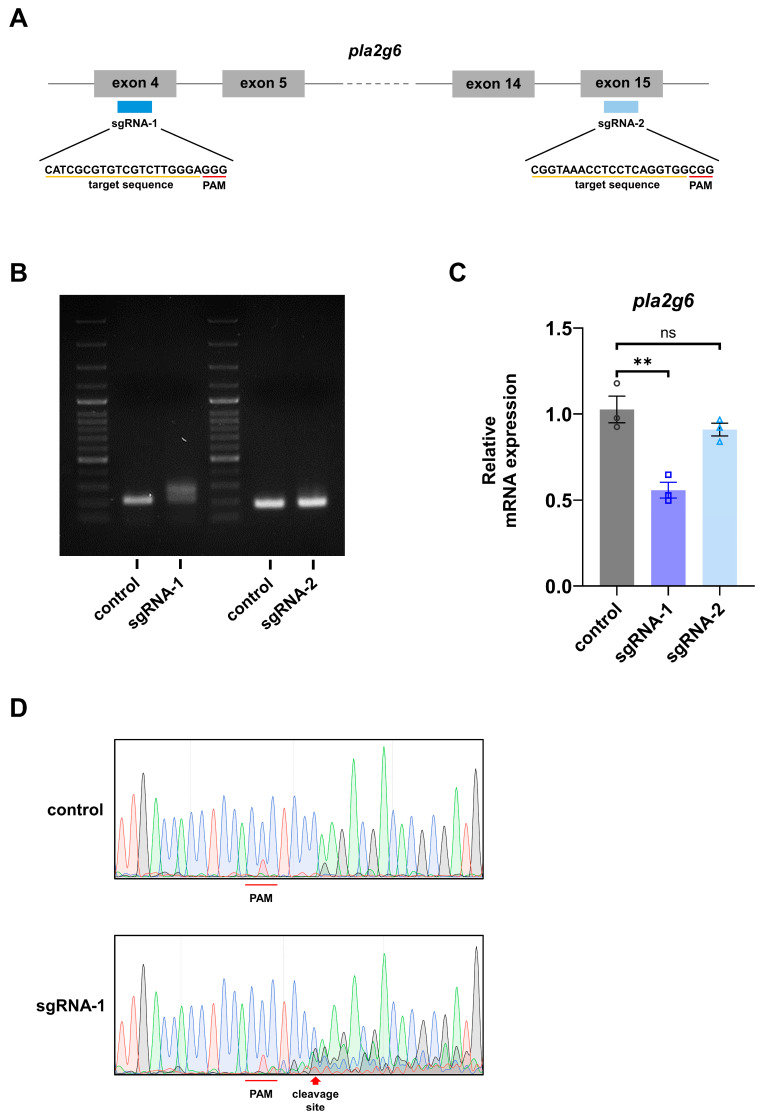
Transient CRISPR/Cas9-mediated disruption of endogenous *pla2g6* in zebrafish embryos. (**A**) Schematic illustration of the zebrafish *pla2g6* genomic locus showing the positions and sequences of two independent single-guide RNAs (sgRNAs) targeting non-overlapping regions of the coding sequence. (**B**) PCR analysis of genomic DNA from zebrafish embryos confirmed targeting of the *pla2g6* locus. Embryos injected with sgRNA-1/Cas9 displayed a characteristic smear in gel electrophoresis, whereas the sgRNA-2 group showed a single intact band indistinguishable from controls, indicating that sgRNA-1 efficiently induced a heterogeneous population of insertions and deletions (indels) via CRISPR/Cas9-mediated mutagenesis. (**C**) Quantitative RT-PCR analysis showed a significant reduction in *pla2g6* transcript levels in embryos injected with sgRNA-1/Cas9 compared with controls, whereas no reduction was observed in embryos treated with sgRNA-2. Relative mRNA expression was quantified by qRT-PCR and normalized to *gapdh*. (**D**) Sanger sequencing chromatograms of PCR-amplified target regions from control and sgRNA-1/Cas9-injected embryos. Control embryos show uniform sequence traces, whereas sgRNA-1/Cas9-injected embryos display mixed and overlapping peaks downstream of the predicted cleavage site, consistent with heterogeneous indel formation in F0 embryos. Green, red, blue, and black peaks represent “A”, “T”, “C”, and “G”, respectively. Data represent three independent biological replicates with each analyzed in three technical repeats to ensure measurement precision. Data are presented as mean ± SEM. Statistical significance was determined using one-way Analysis of Variance (ANOVA) with Tukey’s pairwise comparison; **, *p* < 0.01; ns, not significant.

To investigate the requirement for endogenous Pla2g6 during neural development, we disrupted Pla2g6 function in zebrafish embryos using CRISPR/Cas9-mediated genome editing. Cas9 protein and *pla2g6*-targeting single-guide RNAs (sgRNAs) were injected at the one-cell stage, and embryos were analyzed during early developmental stages. Two independent sgRNAs targeting non-overlapping regions of the *pla2g6* coding sequence were designed (Figure 2A). Genome editing at the *pla2g6* locus was assessed by PCR amplification of the targeted genomic regions. Gel electrophoresis revealed a distinct smear in embryos injected with sgRNA-1, whereas sgRNA-2 produced a single, intact band indistinguishable from control embryos (Figure 2B). This initial screen suggested that sgRNA-1 successfully induced high levels of CRISPR-mediated mutagenesis, while sgRNA-2 remained ineffective. To evaluate the impact of CRISPR-mediated disruption at the transcript level, quantitative RT-PCR (qRT-PCR) was performed to assess *pla2g6* mRNA levels. sgRNA-1, but not sgRNA-2, significantly reduced *pla2g6* transcript levels (Figure 2C), consistent with the genomic PCR result showing efficient mutagenesis by sgRNA-1 but not sgRNA-2. The reduction observed with sgRNA-1 reflects CRISPR/Cas9-induced alterations that decreased the abundance of detectable *pla2g6* transcripts. We further validated the genomic lesions through Sanger sequencing of the sgRNA-1 targeted site. Sequence traces were uniform upstream of the predicted Cas9 cleavage site but displayed mixed and overlapping peaks downstream (Figure 2D), consistent with heterogeneous insertions and deletions generated by CRISPR/Cas9-mediated mutagenesis and the mosaic disruption of *pla2g6* in F0 embryos. Based on these findings, all subsequent experiments utilized sgRNA-1 to disrupt endogenous *pla2g6* expression.

**Figure 3:**
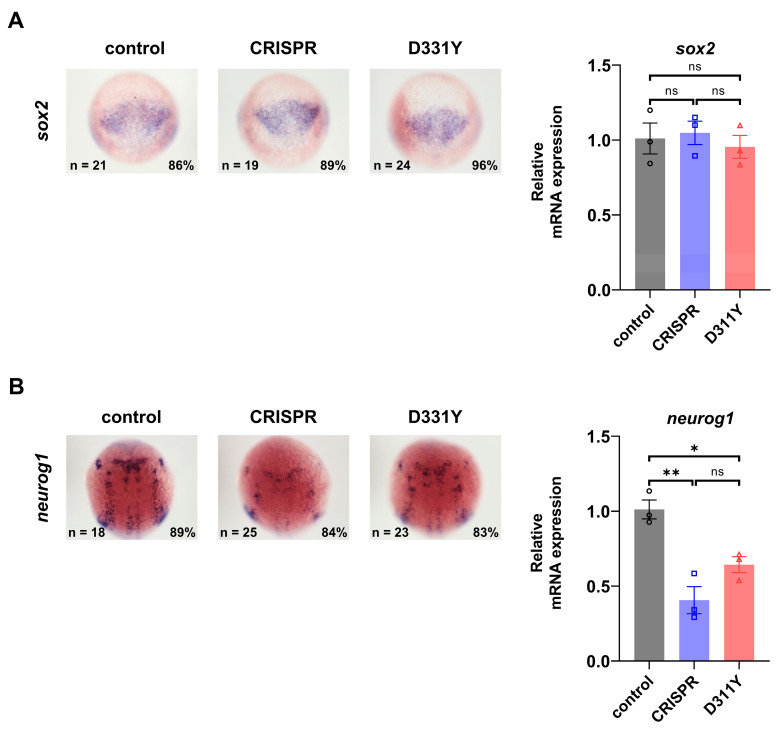
Loss of Pla2g6 selectively reduces neuronal precursors without affecting neural progenitors. Whole-mount in situ hybridization and qRT-PCR were used to assess neural progenitor and neuronal precursor populations in control and *Pla2g6*-deficient zebrafish embryos. (**A**) *sox2* at 8 hpf. Representative in situ hybridization images are shown on the left, with corresponding qRT-PCR measurements of *sox2* mRNA expression on the right. *sox2* expression levels remained comparable across all experimental groups. (**B**) *neurog1* at 10 hpf. Representative images are shown on the left, and qRT-PCR quantification of *neurog1* expression is shown on the right. qRT-PCR analysis demonstrated a significant reduction in *neurog1* expression, with statistical evaluation performed across independent experiments. Both CRISPR-mediated *Pla2g6* deficiency and D331Y-mediated deficiency resulted in a significant reduction in *neurog1* expression. Relative mRNA expression was normalized to *gapdh*. Data are presented as mean ± SEM from three independent biological replicates with each analyzed in three technical repeats. Statistical significance was determined using one-way ANOVA with Tukey’s pairwise comparison; *, *p* < 0.05; **, *p* < 0.01; ns, not significant.

We examined the developing nervous system under conditions of *pla2g6* deficiency using molecular markers expressed at distinct embryonic stages to label specific neural cell populations. Formation of the zebrafish nervous system begins during early gastrulation, around 6 hpf, with neural induction from the ectoderm followed by specification of the neuroectoderm [15,16]. Neural progenitor and stem cells subsequently arise from the neuroectoderm and are characterized by expression of the transcription factor *sox2*, a key regulator of neural progenitor identity [17,18]. Whole-mount in situ hybridization and qRT-PCR showed that the expression pattern of *sox2* was not detectably altered in *pla2g6*-deficient embryos compared with control embryos (Figure 3A), indicating that neural progenitor formation was largely preserved following transient loss of Pla2g6.

Following neural progenitor establishment, progenitor cells differentiate into lineage-restricted neuronal and glial precursors. Neurogenin 1 (*neurog1*, also known as *ngn1*) is expressed in neuronal precursors and is required for neuronal lineage specification, including dopaminergic neuronal precursors [19,20]. To assess whether loss of Pla2g6 affects neuronal precursor formation, we examined *neurog1* expression. At 10 hpf, Pla2g6-deficient embryos exhibited a marked reduction in *neurog1* expression compared with control embryos, as revealed by whole-mount in situ hybridization (Figure 3B). This reduction was further supported by qRT-PCR analysis, which demonstrated a significant decrease in *neurog1* expression (Figure 3B), indicating a reduced number of neuronal precursor cells. Together, these results show that while Pla2g6 is dispensable for neural progenitor establishment, it is required for proper formation or maintenance of neuronal precursors during early neurodevelopment.

To confirm the specificity of CRISPR/Cas-mediated deficiency, a human *PLA2G6* D331Y variant was expressed in zebrafish embryos. This mutation was identified in patients with PARK14 [21] and has been shown to be a loss-of-function mutation that interferes with endogenous PLA2G6 activity and causes PARK14 symptoms in animal models [22,23]. Injection of *PLA2G6* D331Y into zebrafish embryos did not alter *sox2* expression but reduced *neurog1* expression (Figure 3), phenocopying the CRISPR/Cas-mediated phenotype and confirming that the CRISPR/Cas-mediated defect results from disruption of Pla2g6 function. These results validate the specificity of the CRISPR/Cas-mediated phenotype and establish Pla2g6 as a determinant of neuronal precursor formation during early neurodevelopment.

### 2.3. Loss of pla2g6 Induces Apoptosis of Neuronal Precursors Without Affecting Proliferation

**Figure 4:**
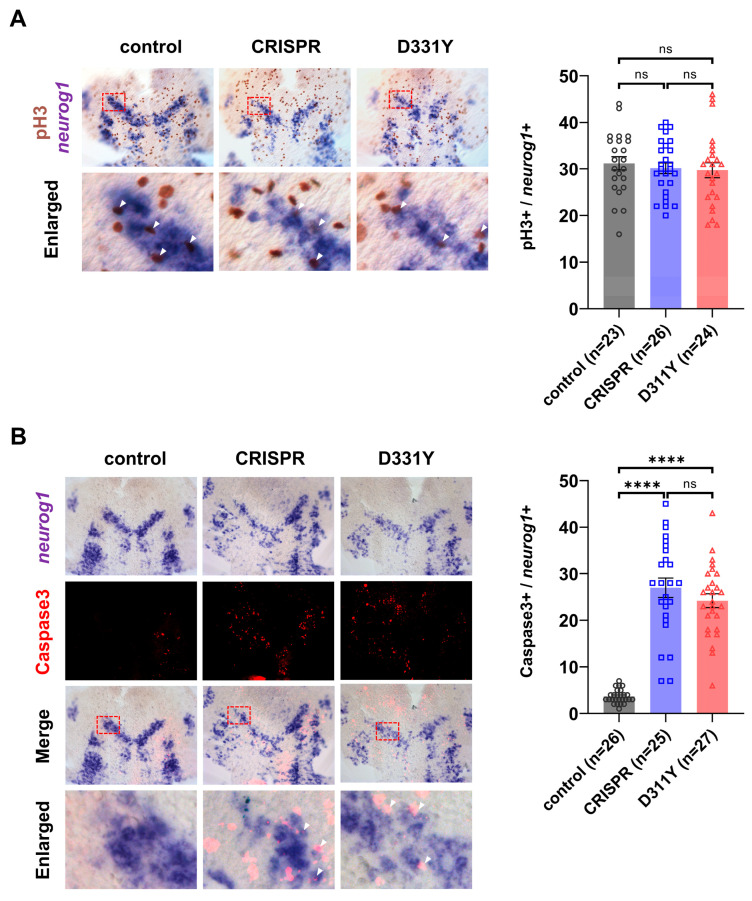
Loss of Pla2g6 induces apoptosis of neuronal precursors without affecting proliferation. Embryos at 10 hpf were flat-mounted after in situ hybridization to facilitate visualization and quantification of signal-positive cells. (**A**) Proliferation analysis. *neurog1* in situ hybridization was combined with immunodetection of phosphorylated histone H3 (pH3). Representative images are shown on the left, with quantification of pH3-positive cells within the *neurog1*-positive region shown on the right. The number of proliferating neuronal precursors was not significantly different between control and Pla2g6-deficient embryos. (**B**) Apoptosis analysis. Co-labeling of *neurog1* transcripts with cleaved caspase-3 identified apoptotic neuronal precursors. Representative images are shown on the left, with quantification of caspase-3-positive cells within the *neurog1*-positive region shown on the right. The bottom rows in panels A and B show enlarged views of the boxed regions (red dashed rectangles), with white arrowheads indicating caspase-3-positive signals. Pla2g6-deficient embryos exhibited a significant increase in apoptotic neuronal precursors compared with controls. Results composed of three independent biological replicates, with 7–10 embryos analyzed per experiment. Individual data points represent cell counts obtained from single embryos. Data are presented as mean ± SEM. Statistical significance was determined using one-way ANOVA with Tukey’s pairwise comparison; ****, *p* < 0.0001 ns, not significant.

To determine the cellular basis underlying the reduction in neuronal precursors observed upon Pla2g6 deficiency, we next examined whether altered cell proliferation or increased apoptosis contributed to this phenotype. We first assessed proliferative activity within the neuronal precursor population. Embryos were subjected to whole-mount in situ hybridization using a *neurog1* riboprobe, followed by immunohistochemical detection of phosphorylated histone H3 (pH3), a marker of cells undergoing mitosis. Quantitative analysis revealed no significant difference in the number of pH3-positive cells within the *neurog1*-positive region between control and Pla2g6-deficient embryos (Figure 4A), indicating that loss of Pla2g6 does not impair proliferation of neuronal precursors.

We then investigated whether increased cell death contributed to the reduced neuronal precursor population. To this end, embryos were labeled for *neurog1* expression and co-stained with an antibody against cleaved caspase-3 to identify apoptotic cells. Pla2g6-deficient embryos exhibited a marked increase in cleaved caspase-3–positive cells within the *neurog1*-positive region compared with control embryos (Figure 4B). Quantification confirmed a significant elevation in neuronal precursor apoptosis following Pla2g6 deficiency. Together, these findings demonstrate that the reduction in neuronal precursors caused by Pla2g6 deficiency is attributable to increased apoptotic cell death rather than impaired proliferative capacity. This suggests that Pla2g6 is required for the survival of neuronal precursors during early neurodevelopment.

### 2.4. Pla2g6 Modulates Neuronal Susceptibility to ROS-Induced Apoptosis During Zebrafish Embryogenesis

**Figure 5:**
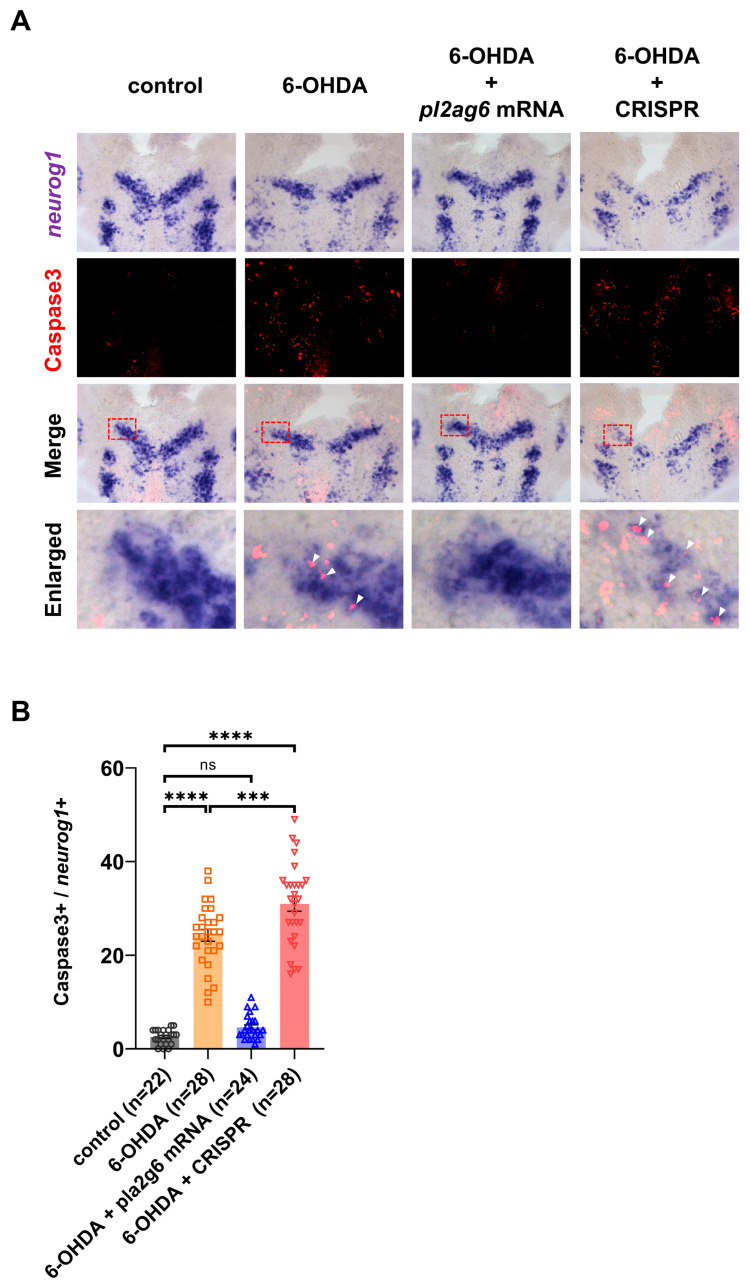
*pla2g6* modulates ROS-induced neuronal apoptosis during zebrafish embryogenesis. The effects of oxidative stress and *pla2g6* manipulation on neural progenitors and neuronal precursors were examined during zebrafish embryonic development. ROS exposure induces apoptosis in the developing nervous system. Embryos treated with 6-hydroxydopamine (6-OHDA) exhibited increased cleaved caspase-3-positive cells within neurog1-positive region compared with untreated controls. To assess the protective role of Pla2g6, one-cell stage embryos were injected with *pla2g6* mRNA and subsequently exposed to 6-OHDA. (**A**) Dual labeling via *neurog1* in situ hybridization and Caspase-3 immunostaining revealed that *pla2g6* overexpression significantly attenuated neuronal apoptosis compared to ROS-treated controls. Transient CRISPR/Cas9-mediated disruption of *pla2g6* increased Caspase3-positive cells following ROS exposure, suggesting that Pla2g6 deficiency sensitizes cells to oxidative stress. Higher-magnification views of the boxed regions are presented in the bottom rows (red dashed rectangles), with white arrowheads marking caspase-3-positive signals. (**B**) Quantification was performed by manually counting apoptotic cells within the *neurog1*-positive region. Data represent three independent biological replicates, with 7–10 embryos per group. Individual data points correspond to cell counts obtained from each embryo. Data are shown as mean ± SEM. Statistical significance was determined using one-way ANOVA with Tukey’s pairwise comparison; ***, *p* < 0.001; ****, *p* < 0.0001; ns, not significant.

Reactive oxygen species (ROS) are important regulators of cellular homeostasis during embryonic development; however, excessive ROS accumulation induces oxidative stress, mitochondrial dysfunction, and apoptosis. Neural tissues are particularly sensitive to oxidative imbalance due to their high metabolic demand and rapid membrane remodeling. Previous studies have shown that PLA2G6 participates in the repair of oxidized phospholipids and protects mitochondria from ROS-mediated damage in mammalian systems [24,25,26]. To determine whether Pla2g6 plays a similar protective role during vertebrate neurodevelopment, zebrafish embryos were exposed to the ROS-inducing compound 6-hydroxydopamine (6-OHDA) during the neural differentiation window. 6-OHDA is widely used to induce oxidative stress through oxidation-dependent redox cycling that generates reactive oxygen species and drives oxidative neuronal damage in vitro and in vivo [27,28,29,30]. 6-OHDA treatment resulted in a marked increase in apoptotic cells within the developing nervous system, as detected by cleaved caspase-3 labeling. Co-localization with neuronal precursor markers indicated that a substantial proportion of apoptotic cells corresponded to developing neuronal populations, demonstrating that elevated ROS levels are sufficient to induce neuronal apoptosis during zebrafish embryonic development (Figure 5). To test whether increased Pla2g6 activity protects developing neurons from oxidative stress, zebrafish embryos were injected with *pla2g6* mRNA at the one-cell stage and subsequently exposed to 6-OHDA. Embryos challenged with oxidative stress exhibited a significant reduction in apoptotic neurons when *pla2g6* was overexpressed compared with ROS-treated controls (Figure 5).

To determine whether endogenous Pla2g6 is required for neuronal resistance to oxidative stress, transient CRISPR/Cas9-mediated disruption of *pla2g6* was performed in F0 embryos. Under basal conditions, Pla2g6-deficient embryos displayed a modest increase in neuronal apoptosis (Figure 4). Following exposure to 6-OHDA, these embryos exhibited a markedly increased number of apoptotic neurons compared with treated control embryos (Figure 5). Quantitative analysis confirmed that loss of Pla2g6 significantly exacerbated ROS-induced neuronal apoptosis (Figure 5). Together, these findings show that Pla2g6 plays a critical role in buffering oxidative stress during early neurodevelopment. Increased Pla2g6 levels protect developing neurons from ROS-induced apoptosis, whereas disruption of Pla2g6 sensitizes neuronal precursors to oxidative damage.

## 3. Discussion

The role of Pla2g6/PLA2G6 in embryonic neural development has not been defined. The present findings in zebrafish embryos identify Pla2g6 as acting at a specific step of neural development, after establishment of the neural progenitor population and during the transition to neuronal precursors, as evidenced by unchanged neural progenitor markers together with reduction in neuronal precursors. This requirement is restricted to a defined developmental window, consistent with enrichment of *pla2g6* expression in the central nervous system during neurogenesis. Rather than influencing proliferative expansion, Pla2g6 is required for neuronal precursor survival, as demonstrated by increased apoptosis in this cell population following Pla2g6 deficiency. These findings identify a stage- and cell type-specific requirement for Pla2g6 during early neurodevelopment.

The increase in neuronal precursor apoptosis observed following Pla2g6 deficiency identifies apoptosis as the primary cellular process underlying the reduction in this cell population during neurogenesis, without changes in proliferation. The mechanism by which Pla2g6 deficiency triggers apoptosis in this context remains to be determined. Previous studies in Drosophila and patient fibroblasts have shown that loss of PLA2G6/iPLA2-VIA function impairs mitochondrial respiratory chain activity, reduces ATP synthesis, causes abnormal mitochondrial morphology, and elevates lipid peroxidation [31]. In *PLA2G6^D331Y/D331Y^* knockin mice, disrupted cristae and mitochondrial dysfunction with upregulated ROS were observed in dopaminergic neurons alongside increased pro-apoptotic gene expression [22]; furthermore, in rotenone-treated SH-SY5Y cells, PARK14 PLA2G6 mutants including D331Y failed to prevent cytochrome c release and activation of the mitochondrial apoptotic pathway [32]. Beyond mitochondrial dysfunction, PLA2G6-derived ceramide generation via neutral sphingomyelinase activation during ER stress has also been shown to trigger intrinsic apoptosis in β-cells [33,34], and reducing elevated ceramide levels in iPLA2-VIA-deficient Drosophila suppressed neurodegeneration [35]. Together, these findings suggest that the apoptosis observed in Pla2g6-deficient embryos may reflect disrupted mitochondrial and lipid homeostasis during a critical stage of neuronal differentiation, though this mechanistic interpretation requires direct experimental validation in the zebrafish model.

In the present study, Pla2g6 overexpression reduces neuronal apoptosis following 6-OHDA-induced ROS exposure, whereas Pla2g6 deficiency markedly enhances ROS-induced apoptosis, indicating that Pla2g6 functions to buffer oxidative stress-triggered apoptotic signaling during neurogenesis. A mechanistic basis for this protective effect is provided by prior studies demonstrating that PLA2G6 repairs oxidized mitochondrial phospholipids, preserves mitochondrial membrane integrity, prevents loss of membrane potential, attenuates cytochrome c release, and reduces ROS accumulation [24,25,26]. Consistent with these protective mechanisms, disease-associated PLA2G6 mutants fail to prevent mitochondrial apoptotic activation in dopaminergic neurons, as discussed above [32]. These findings suggest that, during embryonic neurogenesis where metabolic demand and membrane remodeling are high [36,37], Pla2g6-dependent maintenance of mitochondrial and lipid homeostasis may serve as a critical determinant of neuronal survival under oxidative stress. Although the present study did not directly assess mitochondrial membrane potential, cytochrome c release, or lipid peroxidation in Pla2g6-deficient embryos, the convergence of our functional data with established mechanisms of PLA2G6-mediated mitochondrial protection supports a model in which Pla2g6 safeguards neuronal precursors from ROS-induced apoptosis during a vulnerable window of neural development.

Pla2g6 function during embryonic neurogenesis provides a [potential, hypothetical] developmental context for disorders associated with PLA2G6 mutations. The requirement of Pla2g6 during an early stage of neuronal differentiation suggests that disruption of Pla2g6/PLA2G6 affects neural cell populations prior to maturation. This interpretation aligns with the concept that neurodegenerative disorders involve impaired neurodevelopment in addition to progressive degeneration [38,39,40]. Clinical and genetic studies of PLA2G6-associated disorders further support this view, as conditions such as infantile neuroaxonal dystrophy and PARK14 present with early onset and rapid progression [10,41,42], consistent with the possibility that neuronal vulnerability is established during early stages of nervous system development. These findings raise the possibility that impaired survival of neuronal precursors represents an early cellular event contributing to the pathogenesis of PLA2G6-associated disorders, a question that warrants direct investigation in future studies.

A limitation of this study is that CRISPR/Cas9 disruption was performed in F0 embryos to enable rapid functional assessment during early embryogenesis. This approach results in mosaic mutagenesis, which may introduce variability at the cellular level. We quantified the efficiency of the transient knockout by assessing the proportion of embryos exhibiting the phenotype, and the results revealed consistent phenotypic outcomes across embryos, indicating effective disruption of Pla2g6 function under transient conditions. In addition, the reduction in neuronal precursors and its recapitulation by the loss-of-function PLA2G6 D331Y variant support that the observed phenotype reflects Pla2g6-dependent effects. Another limitation of this study is that a rescue experiment using wild-type Pla2g6 in the context of D331Y expression was not performed, as interpretation of rescue effects is confounded by the presence of endogenous Pla2g6, potential competition among co-expressed proteins, and limited control of expression timing and dosage in the transient system. Despite these limitations, the consistency of the observed phenotypes and the independent validation using the PLA2G6 D331Y variant provide convergent support for the conclusions of this study.

## 4. Materials and Methods

### 4.1. Ethical Statement

All animal procedures were approved by the Institutional Animal Care and Use Committee of Chang Gung University and conducted in accordance with institutional guidelines (IACUC approval numbers: CGU106-066 and CGU109-206).

### 4.2. Zebrafish Husbandry and 6-OHDA Treatment

The Tü (wild-type) zebrafish strain was obtained from the Zebrafish International Resource Center (Eugene, OR, USA). Fish were maintained and bred in a fully equipped aquarium system with semiautomatic stand-alone habitats. Embryos were staged according to hours post-fertilization (hpf) based on established morphological criteria. For oxidative stress experiments, embryos were exposed to 30 μM 6-OHDA from 5 to 10 hpf and collected at 10 hpf for subsequent analysis.

### 4.3. Constructs, cRNA Preparation, and Microinjection

The sequence information for zebrafish *pla2g6* is available under Ensembl: ENSDARG00000060921 and NCBI Reference Sequence: NM_213097.2. The open reading frames of zebrafish *pla2g6* and human *PLA2G6* D331Y were amplified by PCR using 2× Super Hi-Fi Taq PCR MasterMix (BioTools Co., Ltd., New Taipei City, Taiwan ) as described previously [23]. PCR products were subcloned into the pCS2+ plasmid vector (provided by David Turner, University of Michigan, Ann Arbor, MI, USA). Linearized plasmids were used as templates for in vitro transcription. Capped mRNA (cRNA) was synthesized using a standard cap-dependent transcription reaction according to the manufacturer’s instructions.

### 4.4. CRISPR/Cas9-Mediated pla2g6 Disruption

Two independent single-guide RNAs (sgRNAs) targeting non-overlapping regions of the pla2g6 coding sequence were designed. sgRNA-1 targeted exon 4, and sgRNA-2 targeted exon 15. The sgRNA sequences are provided in Figure 2A. For genome editing, sgRNAs were co-injected with Cas9 protein (BioTools Co., Ltd., New Taipei City, Taiwan) into zebrafish embryos at the one-cell stage. Microinjection was performed as described above, and embryos were collected at indicated developmental stages for subsequent analyses.

### 4.5. PCR Amplification and Sanger Sequencing

Genomic DNA was extracted from zebrafish embryos at the indicated developmental stages. Regions flanking the sgRNA target sites within the *pla2g6* locus were amplified by PCR using gene-specific primers (sgRNA-1_Forward: [5′-TTTCGGTTTGTTGTAGGTGTTG-3′] sgRNA-1_Reverse: [5′-CCGCACCTCTTCTCACTAAACT-3′]; sgRNA-2_Forward: [5′-CAGGGTCGTGATGAAGATGTAA-3′] sgRNA-2_Reverse: [5′-CTCACACAATCCACAAGCATCT-3′]). PCR products were analyzed by agarose gel electrophoresis and subsequently subjected to Sanger sequencing (Tri-I Biotech Inc., New Taipei City, Taiwan). Sequencing chromatograms were examined for mixed and overlapping peaks downstream of the predicted Cas9 cleavage sites, indicative of insertions and deletions generated by CRISPR/Cas9-mediated mutagenesis.

### 4.6. Histological Analysis

For whole-mount in situ hybridization, target gene sequences (*pla2g6*, *sox2*, and *neurog1*) were cloned into pGEM-T or pCS2+ vectors and linearized with appropriate restriction enzymes to generate antisense templates. Digoxigenin-labeled RNA probes were synthesized by in vitro transcription using digoxigenin-11-UTP (Roche, Basel, Switzerland; contributor: UNI-ONWARD Corp., New Taipei City, Taiwan) and purified using post-reaction clean-up columns (Sigma-Aldrich, St. Louis, MO, USA; contributor: UNI-ONWARD Corp., New Taipei City, Taiwan) according to the manufacturer’s instructions. Diethylpyrocarbonate-treated water (DEPC-ddH_2_O) was used for all buffer preparation to prevent RNase contamination.

Zebrafish embryos were fixed in 3% paraformaldehyde (PFA) at 4 °C for 16–18 h, followed by dehydration through a methanol gradient (25%, 50%, 75%, and 100% methanol in DEPC-PBST [PBS with 0.1% Tween-20]) and permeabilization at −20 °C for at least 10 h. Hybridization was carried out in HM buffer 1 (50% formamide, 5× SSC [pH 4.5], 0.1% Tween-20, 50 μg/mL *E. coli* tRNA, and 50 μg/mL heparin in DEPC-ddH_2_O) for at least 15 h at 70 °C. Excess probes were removed by three washes in HM buffer 2 (50% formamide, 5× SSC [pH 4.5], 0.1% Tween-20 in DEPC-ddH_2_O), followed by stepwise exchange to SSC buffer (2× to 0.2× SSC) and then to PBST. Embryos were blocked in 5% sheep serum for 1 h at room temperature and incubated with alkaline phosphatase–conjugated anti-digoxigenin antibody (1:5000; Roche, Basel, Switzerland; contributor: UNI-ONWARD Corp., New Taipei City, Taiwan) overnight at 4 °C. After washing in PBST, color development was performed using NBT/BCIP substrate (Roche, Basel, Switzerland; contributor: UNI-ONWARD Corp., New Taipei City, Taiwan) in the dark at room temperature. The reaction was stopped by fixation in 3% PFA.

For immunohistochemistry, embryos were fixed in 3% PFA at 4 °C for 16–18 h and dehydrated through a methanol gradient (25%, 50%, 75%, and 100% methanol in PBST), followed by permeabilization at −20 °C for at least 10 h. Embryos were blocked in 5% goat serum in PBST for 1 h at room temperature and incubated with primary antibodies against cleaved caspase-3 (1:200; 1:200; Abcam, Cambridge, UK; contributor: Asia-Bioscience Co., Ltd., Taipei City, Taiwan) and phosphorylated histone H3 (pH3; 1:300; Merck KGaA, Darmstadt, Germany; contributor: Hong Jin Co., Ltd., New Taipei City, Taiwan) in 2% goat serum in PBST for 2 h. Embryos were then incubated with fluorochrome-conjugated secondary antibodies (1:500; Alexa Fluor 488 or 594 goat anti-mouse IgG; Invitrogen, Carlsbad, CA, USA; contributor: Life Technologies Co., Ltd., Taipei City, Taiwan) in 2% goat serum in PBST at 4 °C for 16–18 h. After washing in PBST (5 min, three times), embryos were post-fixed in 3% PFA for 20 min at room temperature to stabilize fluorescence signals. Embryos were protected from light and mounted in Vectashield mounting medium (Vector Laboratories, Newark, CA, USA; contributor: Asia-Bioscience Co., Ltd., Taipei City, Taiwan) for imaging.

### 4.7. RNA Extraction and Quantitative Real-Time PCR (qRT-PCR) Analysis

Total RNA was extracted from 30–40 zebrafish embryos using TRIzol reagent (Invitrogen, Carlsbad, CA, USA; contributor: Life Technologies Co., Ltd., Taipei City, Taiwan). RNA was purified by phenol–chloroform extraction and reverse transcribed into cDNA using the First Strand cDNA Synthesis Kit (Bionovas Biotechnology Co., Ltd., Seattle, WA, USA; contributor: WonWon Biotechnology Co., Ltd., Taoyuan City, Taiwan). Quantitative real-time PCR was performed using an ABI StepOne™ system with SYBR Green fluorescent dye (ABclonal, Woburn, MA, USA; contributor: BioTools Co., Ltd., New Taipei City, Taiwan). Gene expression levels were normalized to *gapdh* and analyzed by the 2^−ΔΔCt^ method.

### 4.8. Statistical Analysis

Immunohistochemistry-positive cells were quantified manually based on fluorescent signals. Quantification was performed in a blinded manner, with group identities concealed from the investigator during analysis. All statistical analyses were performed using GraphPad Prism (GraphPad Prism 9.5.1, GraphPad Software, La Jolla, CA, USA). Data are presented as mean ± standard error of the mean (SEM). One-way analysis of variance (ANOVA) was performed with a 95% confidence interval, followed by Tukey’s multiple comparisons test. Each experiment was performed with at least two technical replicates. All quantitative data, including cell counts and qRT-PCR measurements, were subjected to statistical analysis as described above.

## 5. Conclusions

In conclusion, this study defines a previously uncharacterized role of Pla2g6 during early neurogenesis by identifying neuronal precursor survival as the critical process dependent on Pla2g6 function. Selective loss of neuronal precursors occurs without changes in neural progenitor formation or proliferation and is driven by increased apoptosis, establishing a stage- and cell type-specific requirement for Pla2g6 during neural lineage progression. Pla2g6 modulates neuronal responses to oxidative stress, linking lipid remodeling and mitochondrial integrity to the control of intrinsic apoptotic signaling during neurodevelopment. These findings provide a developmental framework for PLA2G6-associated disorders and position impaired neuronal precursor survival as a potential early cellular event in disease onset.

## Data Availability

The data that support the findings of this study are available from the corresponding author upon reasonable request.

## Abbreviations

The following abbreviations are used in this manuscript:

ANOVA	Analysis of variance
cRNA	Capped RNA
CRISPR	Clustered regularly interspaced short palindromic repeats
DEPC	Diethylpyrocarbonate
dNTP	Deoxyribonucleotide triphosphate
DTT	Dithiothreitol
hpf	Hours post-fertilization
INAD	Infantile neuroaxonal dystrophy
iPLA2β	Calcium-independent phospholipase A2β
NBIA	Neurodegeneration with brain iron accumulation
PARK14	PLA2G6-associated parkinsonism
PBST	Phosphate-buffered saline with Tween-20
PFA	Paraformaldehyde
PLA2G6	Phospholipase A2 group VI
qRT-PCR	Quantitative real-time polymerase chain reaction
ROS	Reactive oxygen species
RT-PCR	Reverse transcription polymerase chain reaction
SEM	Standard error of the mean
sgRNA	Single-guide RNA
SSC	Saline-sodium citrate buffer

## References

[1] Tang J., Kriz R.W., Wolfman N., Shaffer M., Seehra J., Jones S.S. (1997). A novel cytosolic calcium-independent phospholipase A2 contains eight ankyrin motifs. J. Biol. Chem..

[2] Wolf M.J., Gross R.W. (1996). Expression, purification, and kinetic characterization of a recombinant 80-kDa intracellular calcium-independent phospholipase A2. J. Biol. Chem..

[3] Larsson Forsell P.K., Kennedy B.P., Claesson H.E. (1999). The human calcium-independent phospholipase A2 gene multiple enzymes with distinct properties from a single gene. Eur. J. Biochem..

[4] Ma Z., Wang X., Nowatzke W., Ramanadham S., Turk J. (1999). Human pancreatic islets express mRNA species encoding two distinct catalytically active isoforms of group VI phospholipase A2 (iPLA2) that arise from an exon-skipping mechanism of alternative splicing of the transcript from the iPLA2 gene on chromosome 22q13.1. J. Biol. Chem..

[5] Larsson P.K., Claesson H.E., Kennedy B.P. (1998). Multiple splice variants of the human calcium-independent phospholipase A2 and their effect on enzyme activity. J. Biol. Chem..

[6] Deng X., Yuan L., Jankovic J., Deng H. (2023). The role of the PLA2G6 gene in neurodegenerative diseases. Ageing Res. Rev..

[7] Turk J., Ramanadham S. (2004). The expression and function of a group VIA calcium-independent phospholipase A2 (iPLA2beta) in beta-cells. Can. J. Physiol. Pharmacol..

[8] Gregory A., Westaway S.K., Holm I.E., Kotzbauer P.T., Hogarth P., Sonek S., Coryell J.C., Nguyen T.M., Nardocci N., Zorzi G. (2008). Neurodegeneration associated with genetic defects in phospholipase A(2). Neurology.

[9] Khateeb S., Flusser H., Ofir R., Shelef I., Narkis G., Vardi G., Shorer Z., Levy R., Galil A., Elbedour K. (2006). PLA2G6 mutation underlies infantile neuroaxonal dystrophy. Am. J. Hum. Genet..

[10] Morgan N.V., Westaway S.K., Morton J.E., Gregory A., Gissen P., Sonek S., Cangul H., Coryell J., Canham N., Nardocci N. (2006). PLA2G6, encoding a phospholipase A2, is mutated in neurodegenerative disorders with high brain iron. Nat. Genet..

[11] Magrinelli F., Mehta S., Di Lazzaro G., Latorre A., Edwards M.J., Balint B., Basu P., Kobylecki C., Groppa S., Hegde A. (2022). Dissecting the Phenotype and Genotype of PLA2G6-Related Parkinsonism. Mov. Disord..

[12] Paisan-Ruiz C., Bhatia K.P., Li A., Hernandez D., Davis M., Wood N.W., Hardy J., Houlden H., Singleton A., Schneider S.A. (2009). Characterization of PLA2G6 as a locus for dystonia-parkinsonism. Ann. Neurol..

[13] Engel L.A., Jing Z., O’Brien D.E., Sun M., Kotzbauer P.T. (2010). Catalytic function of PLA2G6 is impaired by mutations associated with infantile neuroaxonal dystrophy but not dystonia-parkinsonism. PLoS ONE.

[14] Polster B., Crosier M., Lindsay S., Hayflick S. (2010). Expression of PLA2G6 in human fetal development: Implications for infantile neuroaxonal dystrophy. Brain Res. Bull..

[15] Kimmel C.B., Ballard W.W., Kimmel S.R., Ullmann B., Schilling T.F. (1995). Stages of embryonic development of the zebrafish. Dev. Dyn..

[16] Wilson S.I., Edlund T. (2001). Neural induction: Toward a unifying mechanism. Nat. Neurosci..

[17] Graham V., Khudyakov J., Ellis P., Pevny L. (2003). SOX2 functions to maintain neural progenitor identity. Neuron.

[18] Lefebvre V., Dumitriu B., Penzo-Mendez A., Han Y., Pallavi B. (2007). Control of cell fate and differentiation by Sry-related high-mobility-group box (Sox) transcription factors. Int. J. Biochem. Cell Biol..

[19] Jeong J.Y., Einhorn Z., Mercurio S., Lee S., Lau B., Mione M., Wilson S.W., Guo S. (2006). Neurogenin1 is a determinant of zebrafish basal forebrain dopaminergic neurons and is regulated by the conserved zinc finger protein Tof/Fezl. Proc. Natl. Acad. Sci. USA.

[20] Ma Q., Chen Z., del Barco Barrantes I., de la Pompa J.L., Anderson D.J. (1998). neurogenin1 is essential for the determination of neuronal precursors for proximal cranial sensory ganglia. Neuron.

[21] Lu C.S., Lai S.C., Wu R.M., Weng Y.H., Huang C.L., Chen R.S., Chang H.C., Wu-Chou Y.H., Yeh T.H. (2012). PLA2G6 mutations in PARK14-linked young-onset parkinsonism and sporadic Parkinson’s disease. Am. J. Med. Genet. B Neuropsychiatr. Genet..

[22] Chiu C.C., Lu C.S., Weng Y.H., Chen Y.L., Huang Y.Z., Chen R.S., Cheng Y.C., Huang Y.C., Liu Y.C., Lai S.C. (2019). PARK14 (D331Y) PLA2G6 Causes Early-Onset Degeneration of Substantia Nigra Dopaminergic Neurons by Inducing Mitochondrial Dysfunction, ER Stress, Mitophagy Impairment and Transcriptional Dysregulation in a Knockin Mouse Model. Mol. Neurobiol..

[23] Yeh T.H., Liu H.F., Chiu C.C., Cheng M.L., Huang G.J., Huang Y.C., Liu Y.C., Huang Y.Z., Lu C.S., Chen Y.C. (2021). PLA2G6 mutations cause motor dysfunction phenotypes of young-onset dystonia-parkinsonism type 14 and can be relieved by DHA treatment in animal models. Exp. Neurol..

[24] Seleznev K., Zhao C., Zhang X.H., Song K., Ma Z.A. (2006). Calcium-independent phospholipase A2 localizes in and protects mitochondria during apoptotic induction by staurosporine. J. Biol. Chem..

[25] Song H., Wohltmann M., Tan M., Ladenson J.H., Turk J. (2014). Group VIA phospholipase A2 mitigates palmitate-induced beta-cell mitochondrial injury and apoptosis. J. Biol. Chem..

[26] Zhao Z., Zhang X., Zhao C., Choi J., Shi J., Song K., Turk J., Ma Z.A. (2010). Protection of pancreatic beta-cells by group VIA phospholipase A(2)-mediated repair of mitochondrial membrane peroxidation. Endocrinology.

[27] Drechsel D.A., Patel M. (2008). Role of reactive oxygen species in the neurotoxicity of environmental agents implicated in Parkinson’s disease. Free Radic. Biol. Med..

[28] Cohen G., Heikkila R.E. (1974). The generation of hydrogen peroxide, superoxide radical, and hydroxyl radical by 6-hydroxydopamine, dialuric acid, and related cytotoxic agents. J. Biol. Chem..

[29] Lotharius J., Dugan L.L., O’Malley K.L. (1999). Distinct mechanisms underlie neurotoxin-mediated cell death in cultured dopaminergic neurons. J. Neurosci..

[30] Pantic I., Cumic J., Skodric S.R., Dugalic S., Brodski C. (2021). Oxidopamine and oxidative stress: Recent advances in experimental physiology and pharmacology. Chem. Biol. Interact..

[31] Kinghorn K.J., Castillo-Quan J.I., Bartolome F., Angelova P.R., Li L., Pope S., Cochemé H.M., Khan S., Asghari S., Bhatia K.P. (2015). Loss of PLA2G6 leads to elevated mitochondrial lipid peroxidation and mitochondrial dysfunction. Brain.

[32] Chiu C.C., Yeh T.H., Lu C.S., Huang Y.C., Cheng Y.C., Huang Y.Z., Weng Y.H., Liu Y.C., Lai S.C., Chen Y.L. (2017). PARK14 PLA2G6 mutants are defective in preventing rotenone-induced mitochondrial dysfunction, ROS generation and activation of mitochondrial apoptotic pathway. Oncotarget.

[33] Ramanadham S., Ali T., Ashley J.W., Bone R.N., Hancock W.D., Lei X. (2015). Calcium-independent phospholipases A2 and their roles in biological processes and diseases. J. Lipid Res..

[34] Lei X., Zhang S., Emani B., Barbour S.E., Ramanadham S. (2010). A link between endoplasmic reticulum stress-induced beta-cell apoptosis and the group VIA Ca^2+^-independent phospholipase A2 (iPLA2beta). Diabetes Obes. Metab..

[35] Lin G., Lee P.T., Chen K., Mao D., Tan K.L., Zuo Z., Lin W.W., Wang L., Bellen H.J. (2018). Phospholipase PLA2G6, a Parkinsonism-Associated Gene, Affects Vps26 and Vps35, Retromer Function, and Ceramide Levels, Similar to α-Synuclein Gain. Cell Metab..

[36] Huang S.H., Huang K.S., Yu C.H., Gong H.Y. (2013). Metabolic profile analysis of a single developing zebrafish embryo via monitoring of oxygen consumption rates within a microfluidic device. Biomicrofluidics.

[37] Zhao X., Chen J., Zhang W., Yang C., Ma X., Zhang S., Zhang X. (2019). Lipid Alterations during Zebrafish Embryogenesis Revealed by Dynamic Mass Spectrometry Profiling with C=C Specificity. J. Am. Soc. Mass Spectrom..

[38] Cheron J., Ranga A., Bonnefont J. (2026). Are neurodegenerative diseases late-onset neurodevelopmental disorders? Tracing the developmental origins of neuronal vulnerability. Front. Neurosci..

[39] Mehler M.F., Gokhan S. (2000). Mechanisms underlying neural cell death in neurodegenerative diseases: Alterations of a developmentally-mediated cellular rheostat. Trends Neurosci..

[40] Schor N.F., Bianchi D.W. (2021). Neurodevelopmental Clues to Neurodegeneration. Pediatr. Neurol..

[41] Shi C.H., Tang B.S., Wang L., Lv Z.Y., Wang J., Luo L.Z., Shen L., Jiang H., Yan X.X., Pan Q. (2011). PLA2G6 gene mutation in autosomal recessive early-onset parkinsonism in a Chinese cohort. Neurology.

[42] Yoshino H., Tomiyama H., Tachibana N., Ogaki K., Li Y., Funayama M., Hashimoto T., Takashima S., Hattori N. (2010). Phenotypic spectrum of patients with PLA2G6 mutation and PARK14-linked parkinsonism. Neurology.

